# On the Radical Identity of the Variolous and Vaccine Forms of Disease

**Published:** 1806-01

**Authors:** Robert Kinglake

**Affiliations:** Taunton


### On the radical Identity of the Variolous and Vaccine Forms of Disease.

*To the Editors of the Medical and Physical Journal.*

Gentlemen,

In a late Number of your Journal* is an extract of a letter from Dr. Sims, containing a remark, that he (the Doctor) had in a paper read to the Medical Society of London, given his opinion, "that the cow-pock is the small-pox of mankind translated to the cow by means of a species of inoculation, and afterwards re-inserted into the human body in a mitigated form." Is this opinion founded on mere conjecture, or is it authorised by the result of actual experiment? About three years since (as several of my friends can testify) a similar opinion occurred to me as being highly reasonable, but as its validity was so directly capable of an experimental test, it appeared to me premature to submit it to the public before subjecting it to that most decisive mode of investigation. A heifer, about two years old, was therefore procured, into apertures on the teats of which made by a dissecting knife, were introduced dossils of lint copiously imbrued with variolous matter, taken from a case of natural small-pox, at the most contagious period of the disease. The dossils were kept in contact with every part of the cut surface sufficiently long, it may be presumed, to have produced the specific impression if that could have been effected. The sores were examined on the third day after the operation, when a slight degree of inflammatory redness and tension was apparent on the edges of the incised parts, but certainly not more than must necessarily have arisen from simple incision. On the fourth,

---

* See No. 81, p. 480.

fourth, fifth, and sixth days the redness and swelling gradually abated, and in a few days afterwards the openings were closed up by a dry eschar, which finally scaled off in about a fortnight. No change whatever occurred in the healed aspect of the inoculated teats after a lapse of several weeks, when, from an opinion that the matter employed might not have possessed its due measure of specific virulence, recourse was had to a fresh portion from another subject, taken from a variolous pustule at an early stage of suppurative inflammation. This was inserted by means of a lancet into the teats of the same heifer, in a manner similar to the common mode of inoculating the human subject. Several horizontal incisions were made in each teat, and in retracting the lancet, care was taken completely to detach the matter from its surface by suitable pressure. On the third day after the insertion there was scarcely any perceptible redness or swelling; on the fourth and fifth still less, and on the tenth the incisions were hardly traceable.

Thus, in two instances, on a heifer but two years old, and which had never numbered with a dairy in which either the variolous or vaccine affections could have been communicated, an attempt to import the small-pox infection wholly failed. But this failure did not warrant me in entirely abandoning the opinion which had led me to the trial; it has ever since that time been my intention to renew the experiment in several additional instances, from an anxious wish to ascertain a fact, that would confer on vaccine inoculation an undeniable claim to universal confidence, as decisively preventive of the more virulent form of small-pox.

It is very conceivable that persons, whilst under the variolous disease, might occasionally be employed in milking cows whose teats might present sore surfaces, arising either from scratches by brambles, excoriations from rough handling in the art of milking, or from various other accidental causes equally capable of producing the effect. At those surfaces a free inlet might be afforded to variolous matter from the hands of the infected milker. If in this mode the *original* or *human* virulence of the disease should have been attempered and rendered comparatively mild by the animal influence of the cow, it surely at once furnishes an inestimable amelioration of a severe disease, and a most important physiological hint respecting the practicability of salutarily modifying other contagious diseases by the gramineous and consequently milder natures of other animals.

The infectious vapour of measles, scarlatina, hooping cough, and that of various forms of febrile affection, may likewise perhaps be so beneficially changed in its active virulence by the respiring and other organic agencies of quadruped animals, as to render their presence within the contagious sphere of diseases importantly advantageous. It is indeed highly probable that the vital powers of different animals will variously affect the morbid conditions of infectious matter, and that instead of multiplying the force and extending the influence of a specific kind of distempered fluid, they would diminish its strength and restrain its diffusion, by inducing chemical alterations in its constituent arrangement and active qualities.

If a number of animals, of the same species, be within the reach of any contagious disease, its spread would most likely (excepting in instances of casual insusceptibility to be affected) be extended to the whole; but were this ravage to be checked by the difference of vital influence occurring in different animals, the specific nature of the affection would be changed and the result perhaps prove a mild and comparatively inoffensive disease. This supposition derives some support from the fact of cats domestically associated with dogs during the prevalence of the disease in the latter, usually termed the *distemper*, which, indeed, appears to be an inveterate catarrhal affection, occasionally destroying by inflammatory violence on the mucous membrane of the fauces and trachea. Cats coming in contact with dogs in this disease, acquire the ailment, but in a degree so extremely slight, as scarcely, it may be presumed, in any instance to prove mortal.

The notion that one disease directly destroys the capability of another, appears to be far-fetched, and to rest on no facts that entitle it to unqualified assent. It is much more probable that the diseased influence is modified by difference of vital power, but that in all cases it is fundamentally identical. The opinion of the *grease of horses' heels*, proposed by Dr. Jenner as the origin of cow-pox, is surely too hypothetical to be capable of any support from facts. Conjecture is here stretched to a much greater latitude than in supposing that the hand of the milker, under variolous disease, has occasionally imparted its infection to the teat of the cow, where it has continued as a contagious disease, but under the mild form which at once characterises terises cow-pox, and constitutes the unyielding ground of its preventive efficacy against small-pox.

Dr. Sims does not say that his opinion is founded on any experiment relative to the cow having been successfully inoculated with variolous matter, and that the effects had been afterwards transferred to the human body in the mitigated form of cow-pox. Are any facts on the subject adduced in the Paper read before the London Medical Society, to which the Doctor alludes? If not, the question is peculiarly proper for experimental inquiry, and should not be relinquished until it shall have undergone sufficient examination by that only certain mode of trial.

My own experiments have hitherto proved inefficient, but have not been carried to a length that could afford conclusive evidence against the object of investigation. In other instances, further attempts shall be made by me, and perhaps the public at large would be more usefully employed in putting the question to this sort of test, than in making *hair-breadth* distinctions on the various and often indefinite aspects of exanthematous disease.

If the cow can be infected by small-pox inoculation, any pustular matter, which might be produced, should be inserted into the human subject; the effects of which would directly go to ascertain what claim of radical identity actually subsists between variolous and vaccine disease, under the formal varieties which present in those several appearances of the malady. That some external difference should arise under circumstances of greater or less virulence of the same disease, is a very natural consequence, and is a difference in degree only and not in quality. All diseases afford ample testimony of similar differences in the various degrees of violence with which they occasionally prevail. But this external diversity involves no essential character.

Varietas non mutat speciem,

If the variolous and vaccine diseases should be identified, the Jennerian substitution of the latter for the former will arrive at an enviable pitch of indisputable importance. Its advantages will then be placed beyond doubt, and it will only remain to choose between a greater and less disease; in making which election, neither difficulty nor discord can possibly arise.

I am, &c.

ROBERT KINGLAKE.

*Taunton, Dec, 12, 1805.*

